# Outcome measurement of extensive implementation of antimicrobial stewardship in patients receiving intravenous antibiotics in a Japanese university hospital

**DOI:** 10.1111/j.1742-1241.2012.02999.x

**Published:** 2012-07-31

**Authors:** T Niwa, Y Shinoda, A Suzuki, T Ohmori, M Yasuda, H Ohta, A Fukao, K Kitaichi, K Matsuura, T Sugiyama, N Murakami, Y Itoh

**Affiliations:** 1 Department of Pharmacy, Gifu University HospitalGifu, Japan; 2 The Center for Nutrition Support & Infection Control, Gifu University HospitalGifu, Japan; 3 Laboratory of Pharmacy Practice and Social Science, Gifu Pharmaceutical UniversityGifu, Japan

## Abstract

**Background:**

Antimicrobial stewardship has not always prevailed in a wide variety of medical institutions in Japan.

**Methods:**

The infection control team was involved in the review of individual use of antibiotics in all inpatients (6348 and 6507 patients/year during the first and second annual interventions, respectively) receiving intravenous antibiotics, according to the published guidelines, consultation with physicians before prescription of antimicrobial agents and organisation of education programme on infection control for all medical staff. The outcomes of extensive implementation of antimicrobial stewardship were evaluated from the standpoint of antimicrobial use density, treatment duration, duration of hospital stay, occurrence of antimicrobial-resistant bacteria and medical expenses.

**Results:**

Prolonged use of antibiotics over 2 weeks was significantly reduced after active implementation of antimicrobial stewardship (2.9% vs. 5.2%, p < 0.001). Significant reduction in the antimicrobial consumption was observed in the second-generation cephalosporins (p = 0.03), carbapenems (p = 0.003), aminoglycosides (p < 0.001), leading to a reduction in the cost of antibiotics by 11.7%. The appearance of methicillin-resistant *Staphylococcus aureus* and the proportion of *Serratia marcescens* to Gram-negative bacteria decreased significantly from 47.6% to 39.5% (p = 0.026) and from 3.7% to 2.0% (p = 0.026), respectively. Moreover, the mean hospital stay was shortened by 2.9 days after active implementation of antimicrobial stewardship.

**Conclusion:**

Extensive implementation of antimicrobial stewardship led to a decrease in the inappropriate use of antibiotics, saving in medical expenses, reduction in the development of antimicrobial resistance and shortening of hospital stay.

**What's known**

- Antimicrobial stewardship programmes are known to promote appropriate use of antibiotics. But, antimicrobial stewardship has not always prevailed in a wide variety of medical institutions in Japan.

**What's new**

- Antimicrobial stewardship intervention was found to be effective in reducing the inappropriate use of antibiotics, shortening hospital stay, reducing the MRSA ratio and saving medical expenses in Japanese hospital.
- Frequent monitoring resulted in an increase in the frequency of recommendation by ICT, reduction in antibiotic consumption and further shortening of antibiotic therapy and hospital stay. These findings supported an importance of day 3 bundle.

## Introduction

Antimicrobial resistance is becoming one of major problems during use of antibiotics worldwide (1,2). It has been demonstrated that inappropriate use of antibiotics is the predominant factor that causes an enhancement of antimicrobial resistance (3,4). Therefore, it is important to prevent or minimise the occurrence of antimicrobial-resistant bacteria. It has been reported that inappropriate use of antibiotics in the hospital ranges from 26% to 57% (5–8). The 12-Step Campaign to Prevent Antimicrobial Resistance Among Hospitalized Adult was established by the Centers for Disease Control and Prevention (CDC), in which withdrawal of inappropriate antibiotics is effective in preventing antimicrobial resistance. Antimicrobial stewardship programmes are known to promote appropriate use of antibiotics (6,9). The Infectious Diseases Society of America (IDSA)/Society for Healthcare Epidemiology of America (SHEA) guidelines recommend two core proactive evidence-based strategies for promotion of antimicrobial stewardship, including ‘formulary restriction and pre-authorization’ and ‘prospective audit with intervention and feedback’ (10,11). The goal of promoting appropriate use of antibiotics is to improve clinical outcomes by reducing the emergence of drug resistance and minimising drug-related adverse events. Furthermore, it has been shown that implementation of antimicrobial stewardship programmes leads to a reduction in the duration of hospital stay and saving in medical expenses (12).

However, such programmes have not always been carried out in a number of medical institutions, where the content of the work of the infection control team (ICT) is confined to the formulary restriction and pre-authorisation on a few specified antibiotics such as carbapenem and antimicrobial agents against methicillin-resistant *Staphylococcus aureus* (MRSA).

In our hospital, we have carried out an extensive intervention programme to optimise antibiotic use since August 2009. The ICT members, including a physician, a clinical pharmacist, a medical technologist and a nurse well trained in infection control, have been involved in the preparation and implementation of the antimicrobial programme. A clinical pharmacist and a physician are mainly in charge of daily review of all prescriptions for inpatients receiving intravenous antimicrobials from a viewpoint of the appropriateness based on the published guidelines.

The aim of the present study was to evaluate the outcomes of the profound implementation of antimicrobial stewardship in the light of the number of inappropriate use, rate of antimicrobial resistance and medical expenses after implementation of the programme.

## Methods

### Ethics statement

The present study was carried out in accordance with the guidelines for the care in human studies adopted by the ethics committee of the Gifu Graduate School of Medicine, and notified by the Japanese government (approval No. 23–175 of the institutional review board).

### Study design

**Figure 1:**
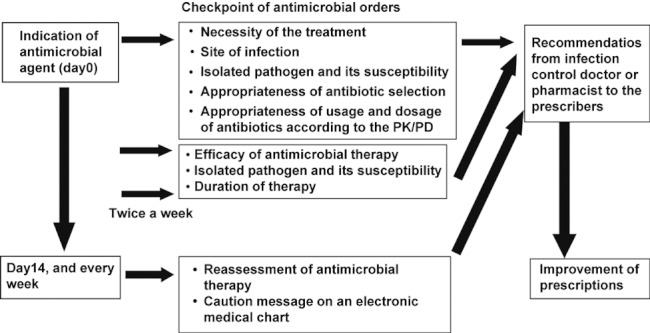
A schema for the review process of antimicrobial orders by infection control team (ICT) members with respect to the usage and dosage of antimicrobial injections, isolated pathogens and site of infection in all inpatients receiving intravenous antibiotics

**Figure 2:**
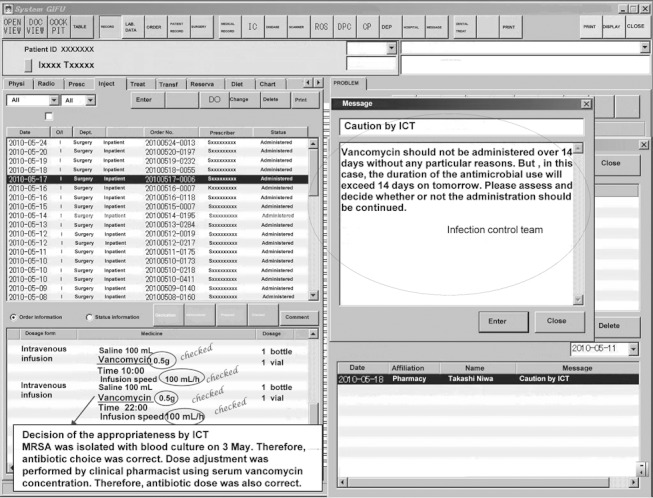
An example of care decision of the appropriate uses of antimicrobial agents using electronic medical chart information and the cautionary message to the prescribers on the electronic medical chart system. Appropriateness of antibiotic selection, usage, dosage was determined by using information of microbiological laboratory results, site of infection, renal function, serum drug concentration, which was obtained by the electronic medical chart. For patients receiving intravenous antibiotics for long periods exceeding 2 weeks, a cautionary message was notified by the ICT member

Our hospital is a national university hospital containing 606 beds. The ICT in our hospital consisted of an infection control doctor, a pharmacist who had a claim on the board-certified infection control pharmacy specialist, a nurse and a microbiological technologist, and has been extensively involved in the implementation of antimicrobial stewardship to all inpatients receiving antibiotic injections since August 2009. Physicians were all informed of the antimicrobial stewardship by ICT members when they prescribed antibiotic injections. The roles of ICT included a review of antimicrobial orders with respect to the usage, dose, isolated pathogens and site of infection for all inpatients receiving parenteral antibiotics, and consultation with physicians before prescription of antibiotics. The review was carried out when the antibiotic injections were prescribed. Patients receiving carbapenem or anti-MRSA agents were reviewed twice a week to facilitate de-escalation therapy. When an inappropriate use of antibiotics was found, ICT members made immediate contact with the prescribers over the telephone (Figure 1). Figure 2 is an example of care decision of the appropriate use of antimicrobial agents using electronic medical chart information. Unless otherwise indicated, the duration of antimicrobial administration was limited within 2 weeks, for patients receiving intravenous antibiotics for a longer period exceeding 2 weeks, a caution message was notified by the ICT members on an electronic medical chart, as shown in Figure 2. However, prolonged use of antibiotic injection over 2 weeks was not regarded as inappropriate for patients with infective septic arthritis (2–4 weeks), endocarditis (4–6 weeks), lung abscess (4–6 weeks) and osteomyelitis (6 weeks). When the message suggesting to discontinue the use was not accepted, ICT members asked the prescriber to stop or change the antibiotics. The appropriateness of antimicrobial use was decided according to the published guidelines, mainly the *Sanford guide to antimicrobial therapy* (13). Appropriateness of duration was also evaluated according to the *Sanford guide to antimicrobial therapy.* Furthermore, the duration was individually evaluated by the infection control doctor and clinical pharmacist. The ICT is also responsible for organising education programme on the topics of hand hygiene in healthcare settings, and antimicrobial therapy such as selection of antibiotics, dosage, treatment duration, 3-day rule and the examples of the inappropriate use of antibiotics for all medical staff twice a year. The ICT also provided the printed information monthly to all medical staff about infection control. Moreover, a physician and a pharmacist are always ready to reply to the inquiries from prescribers about antimicrobial therapy before prescription using mobile phones. Since August 2010, all inpatients receiving intravenous antibiotics were reviewed more than twice a week to enhance the appropriate use of antibiotics, according to the day 3 bundle (14). Furthermore, when antimicrobial injection was started without bacterial culture, the ICT member had started to contact the prescribers to perform bacterial culture (active intervention period). Data were extracted from electronic medical records kept in a central database in our hospital and compared before (period 1; during 1 August 2008 and 31 July 2009) and after (period 2; initial intervention, during 1 August 2009 and 31 July 2010, period 3; active intervention, during 1 August 2010 and 31 July 2011) extensive implementation of antimicrobial stewardship programme.

### Outcomes

The use of antibiotics was converted into defined daily doses (DDDs) per 1000 patient-days, according to the World Health Organization (WHO) guidelines for anatomical therapeutic chemical classification and DDD assignment (15). Only the expenditure of antimicrobial injection was analysed. Prolonged use was defined as the continuous use of intravenous antibiotics over 2 weeks as the indicator of the shortening of the treatment duration. The duration of hospital stay was determined by the Kaplan–Meier plots and the median hospital stay was compared before and after implementation of antimicrobial stewardship using the Mantel–Cox log-rank test. Savings in medical expenses were estimated from the difference in the mean duration of hospital stay before and after intervention and the diagnosis–procedure combination (DPC) of the unit charge of the hospital stay (40% of mean unit charge for hospital stay), and the number of patients receiving antibiotic injections. The exchange rate of 1 dollar was considered as 77.0 Japanese yen.

### Data analysis

Data were analysed using SPSS version 11 (SPSS Inc., Chicago, IL). Parametric variables were analysed using the *t*-test, while non-parametric variables were analysed by the Mann–Whitney U-test or χ^2^ test. p-value of < 0.05 was considered statistically significant.

## Results

### Patient demographics

The patient demographics are shown in Table 1. The annual number of patients receiving intravenous antibiotics was 6251, 6348 and 6507 before implementation (period 1), and after implementation of antimicrobial stewardship (periods 2 and 3), respectively. Although there was no significant difference in gender, slight but significant differences were noted in age and the executing rate of surgical operation.

**Table 1:** Demographics of patients who received antimicrobial injection, antimicrobial consumption, incidence of antibiotic-resistant bacteria before and after implementation of antimicrobial stewardship

	Period 1 before intervention (*n* = 6251)	Period 2 initial intervention (*n* = 6348)	Period 3 active intervention (*n* = 6507)	p-value Period 1 vs. Period 3
Gender (male/female) (*n*)	3334/2917	3417/2931	3466/3041	0.66†
Age (years)*	54 (23.2)	55 (22.5)	56(22.6)	< 0.01††
Operation (with/without) (%)	4140/2111 (66.2%)	4300/2048 (67.7%)	3909/2598 (60.1%)	< 0.01†
**Antibiotic consumption§**				
Penicillins	49.8 (37.8–65.6)	56.4 (34.8–68.5)	47.5 (29.4–62.8)	0.49
First-generation cephalosporins	42.5 (35.9–45.7)	41.7 (36.2–49.8)	44.0 (41.1–47.2)	0.13
Second-generation cephalosporins	13.1 (11.5–18.3)	12.1 (10.9–14.2)	12.0 (9.2–14.4)	0.03
Third-generation cephalosporins	24.2 (18.8–31.7)	25.1 (16.6–34.1)	23.7 (16.1–33.8)	0.82
Fourth-generation cephalosporins	15.7 (9.7–22.9)	15.2 (9.5–22.1)	16.0 (9.9–22.8)	0.73
Carbapenems	27.8 (23.5–44.2)	25.7 (18.1–34.7)	23.3 (15.6–35.1)	0.003
Anti-MRSA agents	13.6 (9.2–19.2)	15.4 (11.3–25.7)	15.5 (10.3–22.9)	0.13
Quinolones	4.6 (1.7–8.6)	4.2 (1.6–8.5)	3.7 (0.4–6.4)	0.09
Aminoglycosides	3.9 (2.4–5.0)	4.0 (1.7–7.5)	1.4 (0.8–2.6)	< 0.001
Others	10.2 (8.1–13.7)	7.6 (6.1–8.6)	4.6 (2.0–6.9)	< 0.001
Total	210.3 (187.8–228.5)	209.3 (165.9–230.6)	192.6 (170.6–208.5)	0.003
Resistant bacteria**				
**No. of patients from whom MRSA was isolated/no. of patients from whom *Staphylococcus aureus* was isolated**	172/361 (47.6%)	152/370 (41.1%)	151/382 (39.5%)	0.026
**No. of patients from whom each pathogen was isolated/no. of patients from whom Gram-negative bacteria were isolated**				
*Acinetobacter baumannii*	59/1026 (5.8%)	67/1024 (6.5%)	56/982 (5.7%)	0.963
*Burkholderia cepacia*	1/1026 (0.1%)	2/1024 (0.2%)	1/982 (0.1%)	0.498
*Citrobacter* species	36/1026 (3.5%)	29/1024 (2.8%)	27/982 (2.7%)	0.329
*Enterobacter cloacae*	81/1026 (7.9%)	92/1024 (9.0%)	64/982 (6.5%)	0.233
*Pseudomonas aeruginosa*	134/1026 (13.1%)	125/1024 (12.2%)	156/982 (15.9%)	0.072
*Serratia marcescens*	38/1026 (3.7%)	30/1024 (2.9%)	20/982 (2.0%)	0.026
*Stenotrophomonas maltophilia*	38/1026 (3.7%)	44/1024 (4.3%)	40/982 (4.1%)	0.668
**No. of patients from whom resistant *P. aeruginosa* was isolated/no. of patients from whom *P. aeruginosa* was isolated**				
Amikacin	1/134 (0.7%)	2/125 (1.6%)	0/156 (0%)	0.939
Ceftazidime	9/134 (6.7%)	6/125 (4.8%)	5/154 (3.2%)	0.784
Imipenem/cilastatin	11/134 (8.2%)	7/125 (5.6%)	15/155 (9.7%)	0.663
Levofloxacin	8/134 (6.0%)	8/125 (6.4%)	8/156 (5.1%)	0.956
Piperacillin	6/134 (4.5%)	3/125 (2.4%)	5/156 (3.2%)	0.797

* Data indicate the mean (standard deviation).

† χ^2^ test

†† Mann–Whitney U-test.

§ Values are antimicrobial use densities expressed as DDD/1000 patient-days, median (range). p-values are for comparisons of period 3 with period 1 by Mann–Whitney U-test.

** Data were statistically compared by χ^2^ test.

### Inappropriate antibiotic use

**Figure 3:**
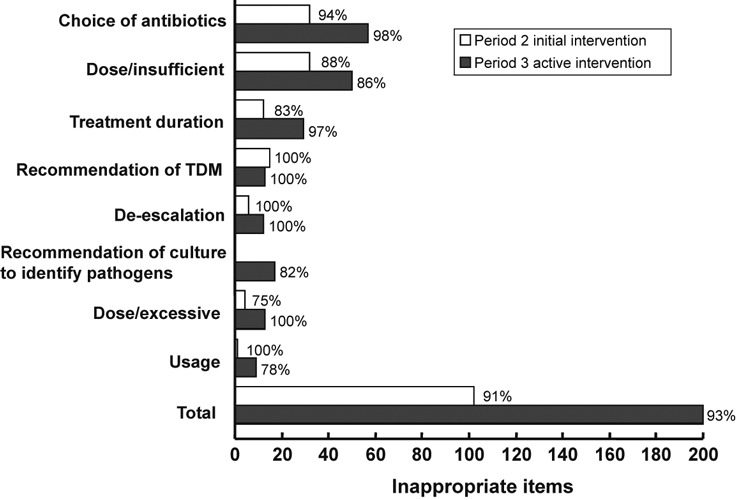
Recommendation contents from the ICT member for prescribers. Percentage values indicate the accepted rate by the prescriber

After implementation of antimicrobial stewardship, a number of inquiries about antimicrobial therapy were made by physicians before prescription (40–50/month). Under such a condition, the ICT members detected 102 cases of inappropriate uses of antibiotics during the initial intervention (period 2). The number of inappropriate uses increased to 200 cases during the active intervention (period 3), in which frequency of review of antimicrobial injections also increased. The items of the inappropriate uses are shown in Figure 3. In such cases, ICT members made proposals on appropriate uses of antibiotics, in which 93 (91%) of 102 proposals in period 2, 186 (93%) of 200 proposals in period 3 were accepted and prescriptions were improved.

### Antimicrobial consumption and treatment duration

**Figure 4:**
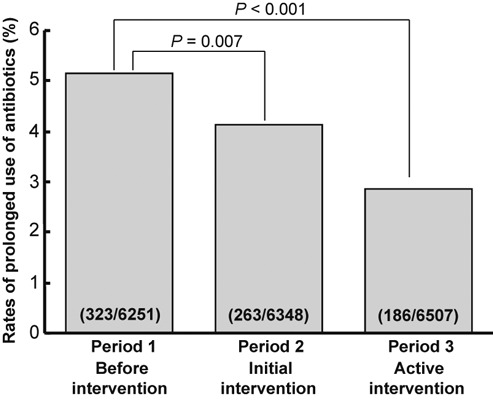
Effect of antimicrobial stewardship intervention on the duration of administration. The rates of prolonged use of antibiotics exceeding 2 weeks were compared before and after intervention

Prolonged use of antibiotics exceeding 2 weeks during period 2 was significantly (p = 0.007) decreased from 5.2% to 4.1% as compared with that during period 1 (Figure 4). The rate of prolonged use of antibiotics was further lowered to 2.9% during the period 3 (p < 0.001 vs. period 1). However, there was no significant difference in the total antimicrobial consumption between period 1 and period 2 [210.3(range: 187.8–228.5) vs. 209.3(165.9–230.6) DDDs/1000 patient-days], although the consumptions of some antibiotics, including second-generation cephalosporins (p = 0.03), carbapenems (p =0.003) and aminoglycosides (p < 0.001), during period 3 were significantly reduced as compared with those during period 1 (Table 1). As a consequence, the total antimicrobial consumption during period 3 was significantly lower than that during period 1 (p = 0.003).

### Changes in occurrence of antimicrobial resistant bacteria

The occurrence of MRSA in the total isolated *S. aureus* significantly decreased after intervention from 47.6% (period 1) to 39.5% (period 3) (p = 0.026) (Table 1). Among patients from whom any Gram-negative rods (GNR) were isolated, the proportion of *Serratia marcescens* was significantly reduced during period 3 as compared with that during period 1 (p = 0.026). In addition, slight and not significant decrease in the occurrence of *Pseudomonas aeruginosa* showing resistance to ceftazidime and piperacillin was observed after implementation of antimicrobial stewardship. However, the rates of resistance to imipenem/cilastatin and levofloxacin were not changed after implementation of antimicrobial stewardship.

### Duration of hospital stay

**Figure 5:**
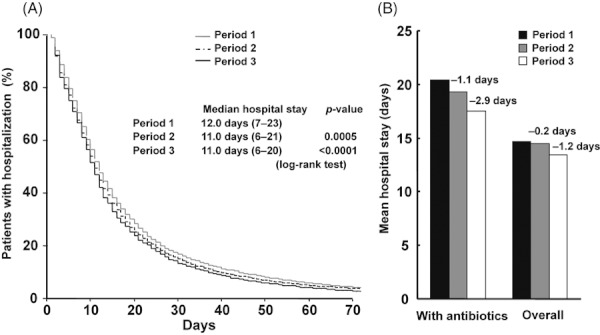
Kaplan–Meier plot for the length of hospital stay who received intravenous antibiotics (A) and comparison of the duration of hospital stay in patients with intervention and in overall patients (B) before and after intervention. The number of patients receiving intravenous antibiotics was 6251, 6348 and 6507 before, initial and active intervention period, respectively, while the number of overall patients was 11,582, 11,570 and 12,321 in each intervention period, respectively. The intervention significantly shortened the duration of hospital stay (log-rank test). Solid line: active intervention period (period 3), dashed line: initial intervention period (period 2), grey line: before intervention period (period 1)

As shown in Figure 5A, Kaplan–Meier plots indicated that the median length of hospital stay was significantly shortened from 12.0 days (interquartile range: 7–23 days) during period 1 to 11.0 days (6–21 days) during period 2 (p = 0.0005 by log-rank test) and 11.0 days (6–20 days) during period 3 (p < 0.0001 vs. period 1). On the other hand, the mean length of hospital stay in patients receiving antibiotic injections was 20.5, 19.3 and 17.5 days during periods 1, 2 and 3, respectively, thereby showing a reduction by 1.1 days during period 2 and by 2.9 days during period 3, although the changes in the duration of hospital stay in all inpatients were only marginal (−0.2 days during period 2 and −1.2 days during period 3) (Figure 5B).

### Saving of cost for antimicrobial injections and medical expenses

Annual cost of antibiotic injection was reduced from US$2.02 million (period 1) to US$2.00 million during period 2 and US$1.86 million during period 3 (Table 2). The costs of antimicrobial injections/patient were US$324 (period 1), US$315 (period 2) and US$286 (period 3), resulting in the savings by 2.8% (US$9/patient) during period 2 and 11.7% (US$38/patient) during period 3. Therefore, the annual savings in antimicrobial cost were estimated to be US$0.058 million during period 2 and US$0.247 million during period 3.

**Table 2:** The analysis of the cost for antimicrobial injections and the total medical expenses in patients receiving antibiotic injection

	Period 1 before intervention	Period 2 initial intervention	Period 3 active intervention
a. Annual cost of antibiotic injection (US$)	2,023,344	1,996,533	1,858,954
b. Number of patients who received antibiotic injection	6251	6348	6507
c. Cost of antibiotic injections/patient (US$) (a/b)	324	315	286
d. Difference in the cost of antibiotic injections/patient (US$, vs. period 1)		9	38
e. Estimated savings of annual cost of antibiotic cost (US$)		58,209	247,253
f. Mean length of hospital stay of patients who received antimicrobial injection (days)	20.4	19.3	17.5
g. Difference in mean hospital stay of patients who received antimicrobial injection (days, vs. period 1)		1.1	2.9
h. Difference in mean hospital stay in all inpatients (days, vs. period 1)		0.2	1.2
i. Difference in shortened duration of hospital stay between patients who received antimicrobial injection and all inpatients (g – h)		0.9	1.7
j. Mean daily hospital charge for the diagnosis–procedure combination (40%) (US$)		341	354
k. Estimated annual saving of medical expenses (US$) (b × i × j)		1,948,201	3,915,913

* Hospital charges included 40% diagnosis–procedure combination (DPC) and 60% fee-for-service basis, and saving of hospital fee was calculated for DPC.

The reduction in the hospital stay (1.0 days in period 2, period 3) was considered to result in considerable savings in medical expenses, in which the amount was estimated to be US$1.95 million in period 2, US$3.92 million in period 3, calculating from the DPC of the mean unit charge for hospital stay (40% of unit charge), and the number of patients receiving antibiotic injections.

## Discussion

The IDSA/SHEA guidelines recommend that prospective audits of antimicrobial use with intervention and feedback to the prescriber can result in a reduction in the inappropriate use of antimicrobials (9). A review and feedback strategy also possesses an educational effect on prescribers. However, this strategy is time-consuming for the reviewer, and is performed mainly by an infectious disease physician or a clinical pharmacist with sufficient experience in infection control. Therefore, the antimicrobial stewardship has not always prevailed in a wide variety of medical institutions in Japan, thereby indicating a gap between the guidelines and clinical practices (16).

To reduce the gap between evidence and clinical practice and to ascertain the clinical outcomes, extensive implementation of antimicrobial stewardship has been carried out since August 2009, which included (i) review of antimicrobial orders by ICT members with respect to the usage, dose, isolated pathogens and site of infection for all inpatients receiving parenteral antibiotics, (ii) consultation with physicians before prescribing antimicrobial agents and (iii) provision of education programme on infection control for all medical staff. The ICT members, particularly, a physician and a pharmacist, organised a co-operative system to accept the inquiries about the choice or usage of antimicrobial agents from prescribers using mobile phones. Indeed, to review all antimicrobial injection is time-consuming. An infectious disease pharmacist was newly placed and consumed almost daytime everyday to review all antimicrobial injections. Subsequently, we evaluated the outcomes of our antimicrobial stewardship programme.

Among 6348 patients who received antibiotic injection, inappropriate use of antibiotics was observed only in 102 cases (1.6%) in period 2. In the active intervention period (period 3), we found 200 cases (3.1%) of inappropriate uses of antibiotics. This rate was much lower than that reported earlier. Kisuule et al. (7) reported that the rate of inappropriate uses of antibiotics was reduced from 57% to 26% after antimicrobial stewardship intervention. Arnold et al. (8) also reported that the rate of inappropriate antimicrobial use reduced from 26% to 7% after intervention. They also showed that the antimicrobial intervention results in fewer recommendations during the intervention period, as the major proportion of orders are already compliant with clinical practice guidelines (17). We did not show the precise rate of inappropriate uses of antibiotics before implementation of antimicrobial stewardship, however, the rate of inappropriate use of antibiotics, as assessed during 1 month before intervention, was 15.1% (data not shown). Consistent with the above report, a marked reduction to 1.6% in the rate of inappropriate use of antimicrobials was attained after intervention in the present study. In our data, the low rate of inappropriate uses detected by ICT members was considered to be due to the following reasons: first, a number of inappropriate uses were assumed to be prevented by the consultation on the proper use of antibiotics from prescribers to ICT members before prescribing. Therefore, we considered that extensive implementation of antimicrobial stewardship led to the optimisation of the antimicrobial prescription before use. Second, even when physicians prescribed with inappropriate uses, clinical pharmacists other than the ICT pharmacist verified the prescription before being checked by ICT members. Third, the introduction of education programme on infection control for all medical staff would draw prescriber’s attention to avoid inappropriate uses. Finally, in our hospital, appropriate uses of antimicrobial agents have been facilitated by the implementation of clinical pathways generated on an electronic medical record system. On the other hand, infection prevention such as hand hygiene has been promoted regardless of the introduction of antimicrobial stewardship. Thus, we considered that promotion of infection prevention had no effect on the improvement of the appearance of antimicrobial resistance in the present study. Among 302 cases in periods 2 and 3, 279 (92%) were accepted and revised, indicating that the proposals by the ICT were adequate. The high rate of acceptance of the proposals was also reported by other investigators (18).

In the present study, the majority of the recommendations about dose adjustment consisted of dose elevation. Evans et al. (19) reported that 50% of patients received excessive dose of antibiotics before antimicrobial intervention. Our data were not consistent with their results. This may be explained by the fact that the approved doses of antibiotics are generally lower in Japan than those recommended by several overseas clinical practice guidelines. For example, we often suggested an elevation of the dose of ampicillin/sulbactam, as the standard daily dose of this agent approved in Japan (6 g/day) is lower than that (12 g/day) approved in western countries.

Several investigators have reported that review and feedback activities reduce antibiotic consumption (20,21). In contrast, Gyssens et al. (22) reported a 25% increase in the antibiotic use after implementation of such interventions in a 948-bed university hospital in the Netherlands. On the other hand, Manuel et al. (23) showed that antimicrobial intervention is associated with a shorter duration of antibiotic therapy, regardless of changes in antimicrobial consumption. In the present study, antimicrobial use density (AUD) was not changed in the initial intervention period (period 2) in spite of shortening of the duration of antibiotic treatment. The lack of change in AUD in period 2 may be due to the fact that the recommendation to elevate the dose of antibiotics was provided in a number of patients. Dose adjustment by elevation of the initial dose may lead to a reduction in the duration of antimicrobial use. However, the active intervention during period 3 caused a significant reduction in the antibiotic consumption with further shortening the duration of antibiotic treatment. In the active intervention period, we consider that frequent monitoring by ICT may facilitate the reassessment of antibiotic therapy and may result in a facilitation of de-escalation or termination of antibiotic therapy.

Several investigators have demonstrated that antimicrobial stewardship results in a reduction in the development of bacterial resistance to antibiotics (21,24,25). In the present study, we surveyed a short-term effect of the present intervention and found that the proportion of MRSA against total isolated *S. aureus* and the proportion of *S. marcescens* against GNR significantly decreased during an active intervention period, although slight and no significant reduction in the antimicrobial-resistant *P. aeruginosa* was observed. However, the occurrence of imipenem-resistant *P. aeruginosa* was reported to be approximately 17% (26) or 21.7% (27) before implementation of antimicrobial stewardship. But, the rates of the appearance of these resistant bacteria were consistently low (< 10%) in our hospital. It has been demonstrated that dose optimisation (28) and reduction in the duration of antimicrobial use (29) are the definite factors that reduce the development of antimicrobial resistance. Therefore, we focused our intervention into optimisation of dose and checking prolonged use of antibiotics.

Dunn et al. (30) reported in a before-and-after study that implementation of antimicrobial stewardship for improvement of the timeliness of switch to oral antimicrobials reduces antimicrobial costs without changing the length of hospital stay. It was noteworthy that, in the present study, Kaplan–Meier plots indicated that median duration of hospital stay was reduced by 1.0 day in periods 2 and 3. It is likely that the present intervention contributes at least in part to the reduction in the hospitalisation period, as the duration of hospital stay in overall patients was not different before and after implementation of antimicrobial stewardship. The reduction in the hospital stay may result in a considerable reduction in medical costs, in which the saving of annual medical expenses was estimated to be US$1.95 million during period 2, and US$3.92 million during period 3. The cost for antibiotic injections used in a patient was also reduced by 2.8% (US$9/patient) in period 2, and 11.7% (US$38/patient) in period 3, indicating that the annual saving of the total cost for antibiotics was US$0.058 million in period 2 and US$0.247 million in period 3.

In the initial intervention, once the prescriptions were reviewed at the start of administration, no verification of the prescriptions was carried out until 2 weeks, except for carbapenem and anti-MRSA agents, although the duration of antimicrobial therapy for 2 weeks is too long in a number of cases. Reassessment of antibiotic prescriptions approximately every 3 days after administration has been shown to be effective for optimising empirical therapy (day 3 bundle) (14). Therefore, in the active intervention period, we carried out more frequent monitoring of antibiotic therapy. We consider that frequent monitoring is especially effective in facilitating the de-escalation or shortening of antibiotic therapy. Indeed, this frequent monitoring resulted in an increase in the frequency of recommendation by ICT, reduction in antibiotic consumption, and further shortening of antibiotic therapy and hospital stay. These findings strongly supported an importance of day 3 bundle. However, frequent monitoring would be difficult to achieve in a number of medical institutions because of the shortage of healthcare professional.

In conclusion, we carried out an extensive antimicrobial stewardship, and the outcomes were evaluated. Our present intervention based on a strategy of antimicrobial stewardship was found to be effective in reducing the inappropriate use of antibiotics, shortening hospital stay, reducing the MRSA ratio, and saving medical expenses in Japanese hospital.
